# A potential function of RLIP76 in the ovarian corpus luteum

**DOI:** 10.1186/s13048-019-0510-8

**Published:** 2019-04-18

**Authors:** Dody Houston Billhaq, Seunghyung Lee

**Affiliations:** College of Animal Life Sciences, Kangwon National University, Chuncheon, 24341 Republic of Korea

**Keywords:** RLIP76, Angiogenesis, Ovary, Corpus luteum

## Abstract

Ral interacting protein of 76 kDa (RLIP76) is multifunctional protein localized and distributed in the plasma membrane, cytosol, and nucleus of the cell. In tumorigenesis, RLIP76 emerges as a common feature for the solid tumor growth. RLIP76 is ubiquitously expressed in various tissues including the ovary. Interestingly, the similar physiological events in obtaining an adequate supply of nutrient by gaining access to the host vascular system are required either for corpus luteum formation or tumor development. In addition, the identical angiogenesis modulators were found in neoplastic and normal ovaries. Our previous study involving RLIP76−/− mice implanted with melanoma or carcinoma cell conclusively demonstrated that RLIP76 is necessary for angiogenesis and neovascularization of primary solid tumors. RLIP76 plays an essential role in tumor angiogenesis through the regulation of pro-angiogenic factors such as vascular endothelial growth factor (VEGF) and hypoxia-inducible factor-1 (HIF-1). In certain previous studies, those pro-angiogenic factors were found significantly to be upregulated during the corpus luteum formation. To that, the following review will discuss the likelihood of RLIP76 role in ovarian corpus luteum.

## Introduction

The female reproductive organ such as ovary is a dynamic tissue that exhibits rapid growth and regression periodically [1]. In an ovarian cycle, the repeated patterns of cellular proliferation, differentiation, and transformation occur during the follicular development and the formation and regression of the corpus luteum [2]. The corpus luteum is a temporary endocrine gland that the major function is producing the progesterone hormone essentially required for establishment and maintenance of pregnancy [3, 4]. During the life span of corpus luteum, this transient reproductive gland undergoes several physiological events including growth, function, and regression [2, 5]. Interestingly, the establishment process of corpus luteum shares identical physiological events with the development mechanism of the tumor in obtaining an adequate supply of nutrient by gaining access to the host vascular system [2, 6]. The similar mechanism of tumorigenesis and ovarian corpus luteum formation inspire us to explore the engagement of Ral interacting protein of 76 kDa (RLIP76), a vital protein for tumor progression, in physiological processes of ovarian corpus luteum.

Two decades ago, the first Ral effector protein which is RLIP76 or Ral binding protein 1 (RalBP1) was discovered by involving the yeast two-hybrid screening system [7, 8]. RLIP76 is a protein harboring multiple domains with numerous functions and appears to be localized and distributed in the membrane, intracellular fluid, and nucleus of the cell [9, 10]. The expression of RLIP76 protein was found in many human tissues including liver, heart, lung, muscle, kidney, and ovary [11]. In tumorigenesis, RLIP76 overexpression emerges as a common feature supporting for the solid tumor growth [12]. In addition, the potential source of such factors locally produced in tumors was also identified in the ovary [13]. Taken together, this review aims to propose the likelihood of RLIP76 role in ovarian corpus luteum.

### RLIP76 is a multifunctional protein

Ral interacting protein of 76 kDa is a modular protein which is capable of interacting with diverse functional proteins [14]. The encoding gene of RLIP76 is located on human chromosome 18p11 [7]. The primary structure of RLIP76 protein constructed by 655 amino acids can be divided into four main regions which are N-terminal region (aa 171–185), Rho-Gap region (aa 210–357), Ral binding region (aa 402–498), and C-terminal region (aa 500–647) [7, 15]. RLIP76 is a vital protein involved in various regulation of cellular and physiological functions through its domain structure as well as adapter sites for multiple signaling proteins [16].

In Ras signaling pathway, Ral-GTPase plays an essential role in a distinct downstream pathway of Ras protein activator [17]. RLIP76, an effector of Ral proteins, appears to participate in endocytosis regulation by binding with several protein partners. In response to epidermal growth factor (EGF) stimulation on a cell surface, protein partners such as RalBP1-associated Eps homology protein 1 (Reps1) and partner of RalBP1 (POB1) bind to C-terminal region of RLIP76 that positively influence on endocytosis and/or cytoskeleton [17, 18]. Both Reps1 and POB1 contain an Eps15 homology domain detected in the EGF receptor substrate [17, 19]. Moreover, an endocytotic machinery protein such as AP2 protein can interact with RLIP76 through its medium chain (μ2) that binds directly on the N-terminal region of RLIP76 [20]. Those protein partners including Resp1, POB1, and AP2 are important for RLIP76 to behave appropriately in endocytosis regulation [20].

In clathrin-dependent endocytosis mechanism, RLIP76 involved in endocytotic machinery as a motor protein appears to be integral with its transport function [10]. RLIP76 has identical function and biochemical properties of dinitrophenyl S-glutathione (DNP-SG) ATPase protein that can catalyze transport of glutathione conjugates and xenobiotic across the biological membrane [21]. RLIP76 is a non-ATP binding cassette (ABC) transporter, but it has two ATP binding sites that exhibit transporting capability of diverse substrates including weakly cationic and anionic compounds [22, 23]. The ATP binding sites of RLIP76 were identified to be ^69^GKKKGK^74^ and ^418^GGIKDLSK^425^ respectively located in the N-terminal and C-terminal region of RLIP76 [22]. In addition, the transport function of RLIP76 plays an important role in the cell stress response mechanism [24]. Furthermore, under the stress condition, RLIP76 mediates the activation of heat shock factor 1 (HSF1) in response to the activation of Ral signal transduction pathway [25].

**Fig. 1:**
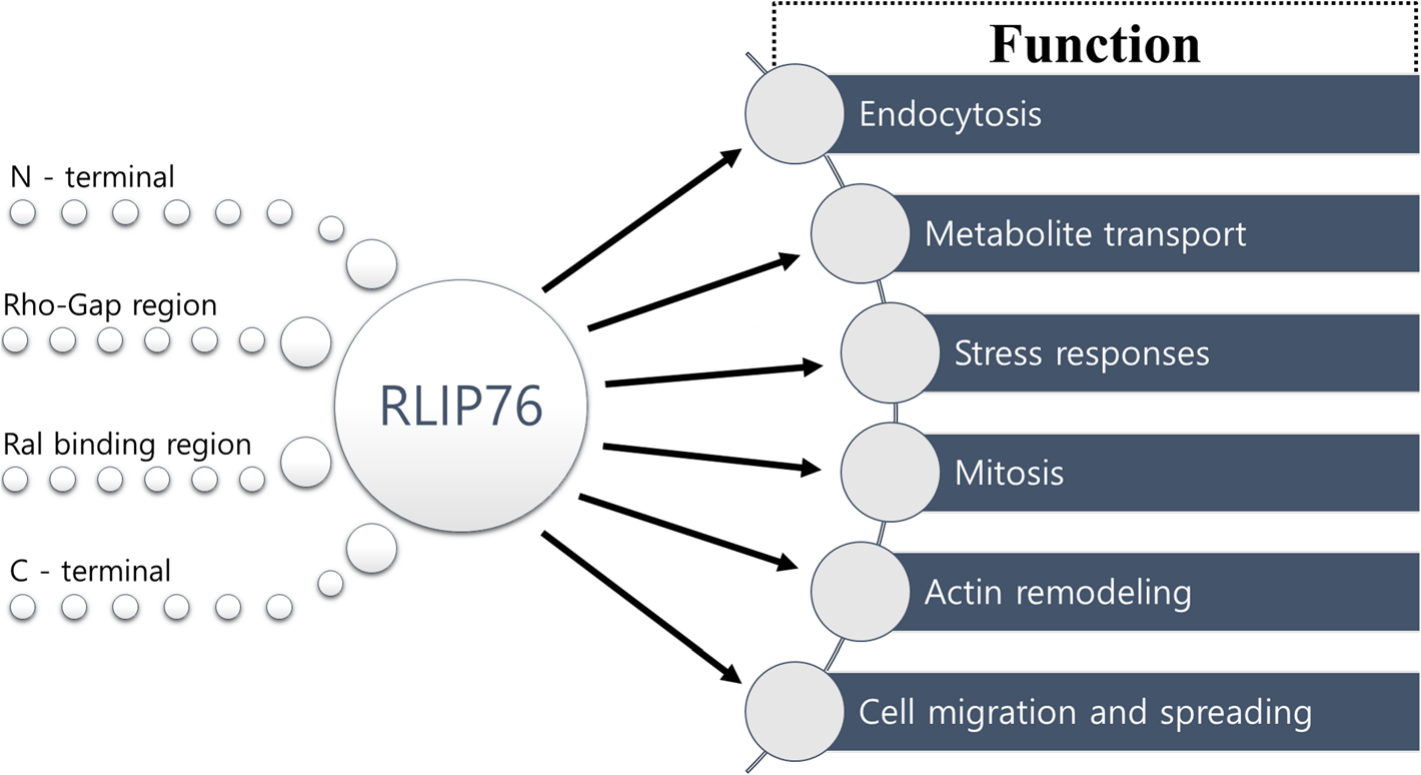
Multiple functions of RLIP76. RLIP76 is a modular protein harboring an N-terminal region, a Rho-Gap region, a Ral binding region, and a C-terminal region. RLIP76 is capable of interacting with diverse functional proteins through its variety of domains and motifs. Thus, RLIP76 is a multifunctional protein involved in various cellular and physiological functions such as endocytosis, metabolite transport, stress responses, mitosis, actin remodeling, cell migration and spreading

In Ral signaling pathway, RLIP76 plays a vital role in mediating numerous cellular processes of small Ras-like GTPase protein associated with its Ral binding region. RLIP76 markedly promotes mitochondrial fission during mitosis through its association with RalA [26]. RLIP76 also acts to regulate actin remodeling due to the activation of RalB induced by FGF (fibroblast growth factor) signal transduction [27]. Moreover, RLIP76 participates in bridging the Ral GTPase to Rho pathways that modulate the activity of CDC42/Rac/Rho Pathway [7]. To that, RLIP76 is critical for adhesion-mediated Rac activation via its Rho-Gap region that regulates cell migration and spreading [28]. Collectively, RLIP76 is involved in various cellular and physiological function such as endocytosis, metabolite transport, stress responses, mitosis, actin remodeling, cell migration and spreading (Fig. 1). Thus, the variety of domains and motifs that belongs to RLIP76 provides heterogeneous ability in cellular function as a multifunctional protein [29].

### Apoptotic function of RLIP76

The presence of RLIP76 appears to be indispensable for cancer cells indicated by its overexpression in various malignant cell lines. Investigations of RLIP76 protein expression level in a number of cells involving detection by Western blot analyses, purification by DNP-SG affinity chromatography, and quantification by enzyme-linked immunosorbent assay (ELISA) were conducted in certain previous studies. The expression of RLIP76 in several malignant cell lines including Caki-2 (human kidney), DG-1 (human melanoma), H358 (human lung, NSCLC), H1618 (human lung, SCLC), OVCAR-3 (human ovary), PC-3 (human prostate), and SW-480 (human colon) were discovered relatively greater amount compared to normal cells [30, 31]. The higher expression of RLIP76 protein in cancer cell lines occurred in line with its greater activity [31]. RLIP76 upregulation in malignant cells is associated with its function to counter apoptosis mechanism that heavily contributes to the cancer cell viability.

In the hierarchy of stress defense mechanisms of cancer cell survival, RLIP76 is an anti-apoptotic protein that occupies a prominent signaling pathway as a glutathione-conjugates (GS-E) and xenobiotic transporter [32]. RLIP76 actively participates in efflux pumping of 4-Hydroxy-t-2,3-nonenal (4HNE) and chemotherapeutic drugs resulted in apoptosis resistance of lung cancer cells [33, 34]. Furthermore, the cytotoxicity of chemotherapeutic drugs mediated apoptosis such as anthracycline (DOX), and vinca-alkaloids (vinblastine, vincristine, and vinorelbine) were relatively low in kidney cancer cells due to RLIP76 overexpression and its transport function [35]. RLIP76 is an ATP-dependent transporter by which its transport activity is provoked as an outcome of phosphorylation by protein kinase C (PKC)-α [36, 37]. The ATPase activity and transport function of RLIP76 remarkably contributes to anti-apoptosis and multidrug resistance (MDR) mechanism in cancer cell [21, 35].

In oxidation, radiation, and heat exposure, RLIP76 also emerges to be vital in the stress response mechanism generating cells resistance to apoptosis. Under mild oxidative stress state, RLIP76 was found to be upregulated on the surface membrane of EAhy926 endothelial cells that contributes to physiologic cellular defenses [38]. RLIP76 also plays a role as a radioprotective agent in which significantly improves the survival duration of mice treated with RLIP76 liposomes exposed by 500-cGy X-irradiation [39]. Moreover, RLIP76 was identified to interact with HSF1 in heterocomplexes [25]. In response to mild transient heat shock exposure, the human cancer cells including K562 (human leukemia), H-226 (human, NSCLC) and H-69 (human, SCLC) incubated with hGST5.8 and RLIP76 produce cells resistance to apoptosis [24].

The blocking or depletion of RLIP76 significantly caused apoptosis in cancer cell lines by which interfere its anti-apoptotic physiological function [40, 41]. RLIP76 plays an important role in apoptosis inhibition by controlling intracellular concentration of pro-apoptotic endogenous lipid peroxidation byproducts through its transport function [24, 42]. In addition, RLIP76 essentially participates in mediating the activation of PI3K/Akt signaling pathway that induces further activation of mTOR which suppresses the expression of the pro-apoptotic constituents [43]. Furthermore, RLIP76 absence appears to be crucial that leads to apoptosis via the activation of the c-Jun kinase (JNK)/stress activated protein kinase and caspase 3 [24]. Thus, RLIP76 is key stress defensive and anti-apoptotic protein that is vitally involved in apoptosis regulation [23].

### Role of RLIP76 in tumor angiogenesis

Angiogenesis is the process of new blood vessel formation originated from the preexisting mature vessel [44]. Multiple regulators, mediators, and signal transduction pathways are involved in the angiogenesis complex process [45, 46]. The pro-angiogenic factors are essential to angiogenesis initiating the proliferation and migration of endothelial cells throughout the neovascularization process [47, 48]. Angiogenesis provides blood supply from the host vascular system that essentially nourishes tumor progression [6].

For a tumor to grow and become malignant, owning blood supply through angiogenesis is heavily required to develop the size beyond 1 mm in diameter [49]. Our previous study involving RLIP76−/− mice implanted with melanoma or carcinoma cell conclusively demonstrated that RLIP76 is necessary for angiogenesis and neovascularization of primary solid tumors [50]. In addition, the blockade of RLIP76 reported in several previous researches led to complete regression in the mice xenograft model of the human lung [32], colon [32], prostate [51], and pancreatic cancer cells [43]. Moreover, angiogenesis is not only fundamentally required for solid tumor development but also metastasis [52].

In tumor growth, the expression of RLIP76 is related to cell migration and is requisite for human cancer cell metastasis [53]. RLIP76 actively participates in cell spreading and migration by regulating Rac1 and Arf6 signaling pathway [54]. In cell mechanism, RLIP76 mediates R-Ras to adhesion-induced Rac activation via a GTPase cascade [28]. The Rho-Gap domain of RLIP76 provides directly binding to R-Ras in a GTP-dependent manner resulted in adhesion-mediated the activation of Arf6 GTPase [28]. In the activation of Arf6, RLIP76 regulates Arf6 via PI3-Kinase-dependent pathway by which RLIP76 contribute to link R-Ras-dependent trisphosphate (PIP3) in the recruitment of a guanine nucleotide exchange factor for Arf6 (ARNO) [54]. The interaction of ARNO with serine residues 29 and 30 of the RLIP76 N-terminus renders the enhancement of Arf6 activation [54]. Subsequently, the activated Arf6 GTPase leads to the promotion of Rac1 GTPase activation [28]. Thus, RLIP76 plays a vital role as a critical link in a small GTPases downstream effect in Rac1 and Arf6 signaling pathway which is essential for tumor angiogenesis.

### RLIP76 is a potential factor in the function of ovarian corpus luteum

The corpus luteum is a heterogenous gland consisted of the large and small luteal cell, endothelial cells, fibroblasts, smooth muscle cells, and immune cells [55]. In the cyclical remodeling of corpus luteum, the regular period of growth, function, and luteolysis occur during the corpus luteum life span [2]. The corpus luteum formation takes place subsequent to ovulation by which constituted of residual follicular wall cells (granulosa and theca cells) [4]. Series of morphological and biochemical changes in cells of theca interna and granulosa of the preovulatory follicles characterize the initiation process of corpus luteum establishment [2]. Certain processes including luteinization of follicular cells, endothelial cell invasion, and tissue remodeling are essential in the establishment of corpus luteum [56].

In the normal functioning of the female reproductive system, angiogenesis plays critical role in the follicular development and establishment of the corpus luteum [13]. The blockade of angiogenesis bears a significant effect in the attenuation of folliculogenesis, the nuisance of ovulation, and the disruption of the development and physiological function of the corpus luteum [57]. Profound angiogenesis takes place in the corpus luteum which is the formation site of a dense capillary network providing the hormone-producing cells for acquiring the oxygen, nutrients, and hormone precursors required for synthesizing and releasing a large number of progesterone hormones [58]. The angiogenesis in corpus luteum characterized by the intense blood vessel formation is frequently compared with angiogenesis in rapidly growing and malignant tumor [4].

An initial presumption that RLIP76 may participate in angiogenesis within the ovary emerge from our previous study result in primary solid tumor neovascularization. RLIP76 plays a critical role in solid tumor progression through angiogenesis derived from the host vascular cells [50]. In the inspection of tumor blood vessel formation in blunted RLIP76 −/− mice, the dimension and density of blood vessel appear to be fewer than wild-type mice. The appearance of the central vessel in the xenografted tumor of wild-type mice was thicker and longer with many branching vessels compared to blunted RLIP76 −/− mice based on 3-dimensional structure produced by the X-ray μCt scanning. The smaller total vascular volume and diameter of tumor in the blunted RLIP76 −/− mice indicated that blockade of RLIP76 impaired efficient neovascularization in implanted solid tumor. RLIP76 plays role independently to the growth of tumor cells and angiogenesis from the host vasculature in solid tumors. In Matrigel in vivo, RLIP76 is necessitated for angiogenesis by which the blood and blood vessel were completely devoid in the Matrigel plugs implanted in blunted RLIP76 −/− mice with or without vascular endothelial growth factor (VEGF). Furthermore, Investigation of cellular mechanism in microvasculature endothelial cells isolated from wild-type and RLIP76 −/− mice in vitro discovered that RLIP76 is required for efficient endothelial cell migration, proliferation, and cord formation fundamentally involved in the angiogenesis process [50].

A strong promoter of angiogenesis, VEGF, plays a critical role for corpus luteum formation and its physiological function in mammalian ovaries [59]. Inhibition of VEGF in a rat model treated with truncated soluble Flt-1 receptors resulted in massive suppression of corpus luteum angiogenesis [60]. In corpus luteum angiogenesis observed in mares, expression of VEGF mRNA and protein were found to be upregulated in the early and mid-luteal phases [61]. Similar result related to the upregulation of VEGF expression during corpus luteum angiogenesis also reported in several previous study in porcine, ovine, bovine, mice and human [62–66].

Our previous study in revealing the mechanism of RLIP76 regulating endothelial cells angiogenic response convinces an insight that RLIP76 may involve in angiogenesis within the ovary. RLIP76 demonstrates a vital role as an angiogenic factor that is required for expression and secretion of VEGF in tumor cells [52]. Transient transfection of RLIP76 small hairpin RNA (shRNA) plasmid into B16F10 mouse melanoma and Lewis lung carcinoma cells resulted in decreasing of VEGF expression level. Melanoma and carcinoma cells transfected with RLIP76 shRNA exhibit incapability to stimulate proliferation and migration of BAEC (Bovine aortic endothelial cells) in conditioned medium in vitro. RLIP76 mainly participates in transactivation of endothelial cells by the tumor secretome through regulating the expression and secretion of angiogenic genes including VEGF in tumor cells [52].

In pro-angiogenic protein regulation, the hypoxia-induced mechanism is significantly involved in promoting the upregulation of VEGF expression [67]. The hypoxia-inducible factor-1 α (HIF-1α) plays a vital role in mediating the expression of VEGF in luteal cell during mammalian corpus luteum establishment through the hypoxia-induced transcriptional mechanism [59]. In a study of ovarian angiogenesis, the mRNA expression of HIF-1 was found to be upregulated in the early luteal phase during corpus luteum formation [64]. Evidently, the emergence of RLIP76 is essential for efficient HIF-1 activation in which depletion of RLIP76 led to a significance blockade of HIF-1 transcriptional activity in both melanoma and carcinoma cell under normoxic conditions [52].

Multiple steps involved in activation of HIF-1 transcription in which posttranslational modifications such as stabilization of HIF-1α subunit, phosphorylation, and nuclear translocation appear to contribute in proper transactivation activity of the HIF-1 regulation [68]. In HIF-1 signaling pathway, PI3K /Akt signal transduction plays a vital role in HIF-1 activation by which PI3-kinase activities are required for induction of HIF-1α protein expression [69]. The previous study of human chorionic gonadotrophin (hCG) role in corpus luteum angiogenesis reported that PI3K/Akt signaling pathway significantly contributes to the induction of VEGF and HIF-1 expression in the luteal cell under both hypoxic and normoxic condition [70]. In a study of pancreatic cancer, RLIP76 plays an important role in PI3K phosphorylation which is essential as an upstream signal signaling node in transducing mitogenic signals related to cell membrane receptors such as growth factors and integrins [43]. Those growth factors and integrins are vitally involved in the angiogenesis signaling pathway [48].

**Fig. 2:**
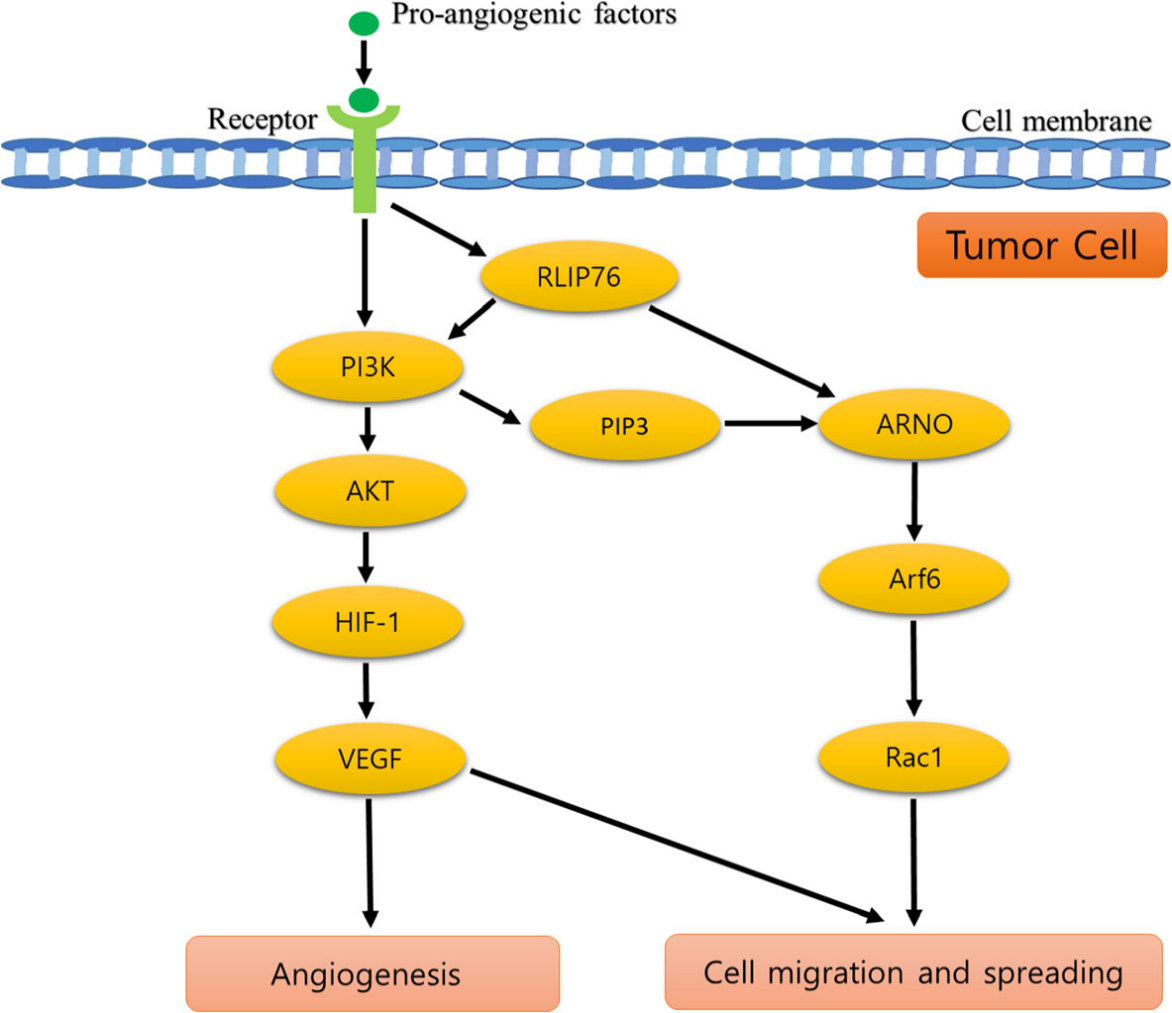
The proposed schematic diagram of RLIP76 role in angiogenesis. In response to pro-angiogenic factors stimulation, RLIP76 mainly participates in mediating the activation of PI3K/Akt signaling pathway that induces further activation of HIF-1. The activation of HIF-1 is required for promoting the VEGF expression that is essential for angiogenesis and cell migration and spreading. In addition, RLIP76 actively participates in regulating Rac1 and Arf6 signaling pathway that heavily contributes to cell spreading and migration. Thus, RLIP76 plays an important role as a critical link in a small GTPases downstream effect in response to membrane transduction of pro-angiogenic factors in which is vital for tumor angiogenesis

**Fig. 3:**
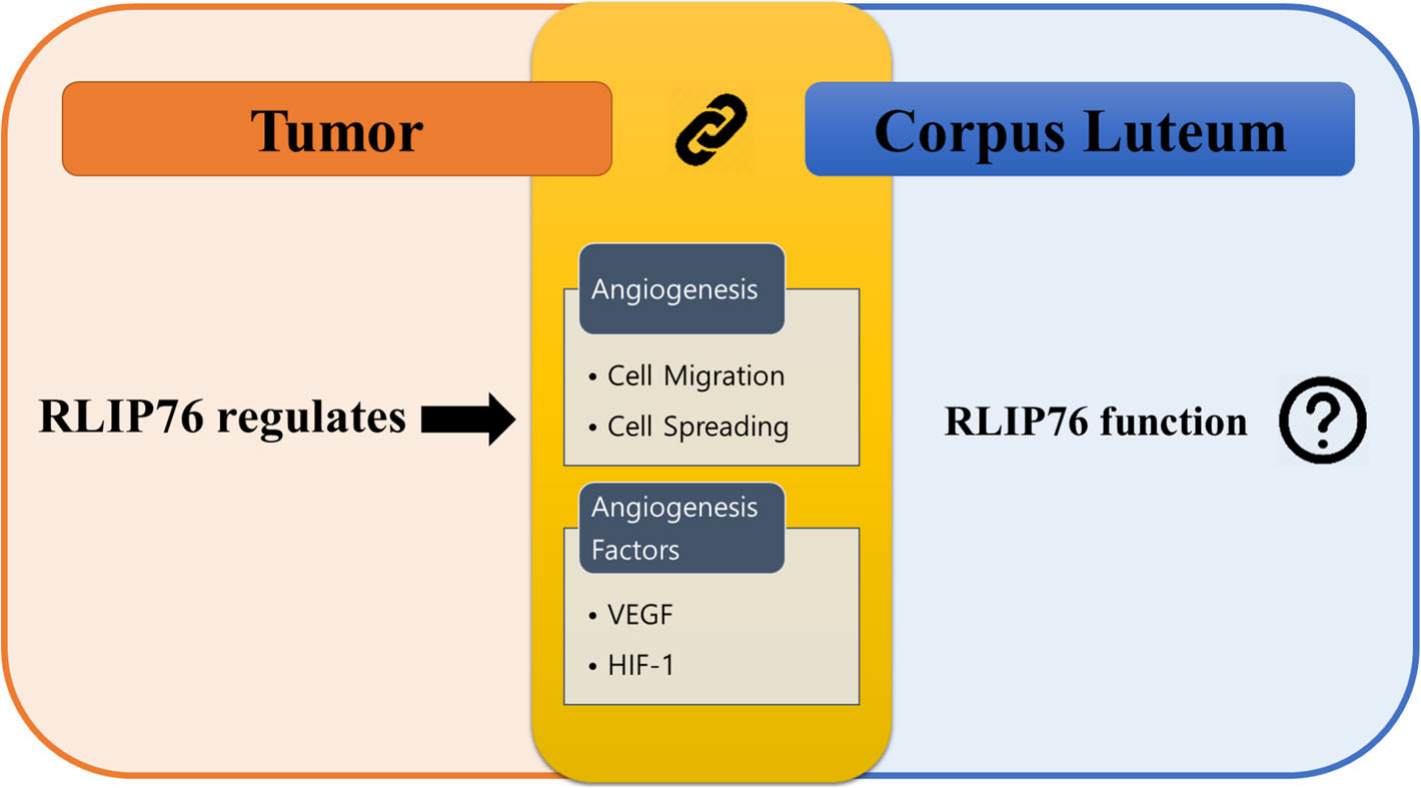
RLIP76 potential function in ovarian corpus luteum. The tumor and corpus luteum share similar physiological events in obtaining an adequate supply of nutrient by gaining access to the host vascular system. In tumor development, RLIP76 actively participates in regulating the cell migration and spreading required for angiogenesis. Furthermore, RLIP76 plays an essential role in tumor angiogenesis through the regulation of pro-angiogenic factors such as VEGF and HIF-1. In addition, those pro-angiogenic factors were discovered to be upregulated during the corpus luteum angiogenesis. Taken together, the RLIP76 may be required for corpus luteum formation and its physiological function. However, further investigation is necessary to discover and elucidate the potential function of RLIP76 in ovarian corpus luteum regulation

In summary, RLIP76 is essential in tumor angiogenesis through the regulation of pro-angiogenic factors such as VEGF and HIF-1 (Fig. 2). RLIP76 is vital protein for tumor progression and is expressed in various tissues including the ovary. Ovarian corpus luteum requires blood supply in order to develop and to function properly. The cellular mechanisms of the normal and tumor ovary were found to be homogeneous throughout the process of neovascularization [45]. Furthermore, the expression pro-angiogenic factors such as VEGF and HIF-1 was discovered to be upregulated during the corpus luteum angiogenesis. Taken together, the RLIP76 may be required for corpus luteum formation and its physiological function (Fig. 3).

## Conclusion

The ovary undergoes dynamic cellular process during the corpus luteum angiogenesis. The angiogenesis is crucial for corpus luteum formation, progression, and its endocrine functions. Interestingly, the neoplastic and normal ovaries secrete identical angiogenesis modulators [45]. The approaches described above related to the RLIP76 role in cellular and molecular events of the tumor have provided a convincing potential function of RLIP76 in ovarian corpus luteum angiogenesis. However, further investigation to elucidate the mechanism and function of RLIP76 in ovarian corpus luteum is necessary for improving our understanding related to the involvement of the oncogenic protein in corpus luteum regulation.

## Abbreviations

4HNE: 4-Hydroxy-t-2,3-nonenal
ABC: ATP binding cassette
ARNO: A guanine nucleotide exchange factor for Arf6
DNP-SG: Dinitrophenyl S-glutathione
EGF: Epidermal growth factor
ELISA: Enzyme-linked immunosorbent assay
FGF: Fibroblast growth factor
GS-E: Glutathione-conjugates
hCG: Human chorionic gonadotrophin
HIF-1α: Hypoxia-inducible factor-1 α
HSF1: Heat shock factor 1
MDR: Multidrug resistance
PIP3: Trisphosphate
PKC: Protein kinase C
POB1: Partner of RalBP1
RalBP1: Ral binding protein 1
Reps1: RalBP1-associated Eps homology protein 1
RLIP76: Ral interacting protein of 76 kDa
VEGF: Vascular endothelial growth factor

## References

[1] Reynolds LP, Killilea SD, Redmer DA (1992). Angiogenesis in the female reproductive system. FASEB J.

[2] Schams D, Berisha B (2004). Regulation of corpus luteum function in cattle - an overview. Reprod Domest Anim.

[3] Niswender GD, Juengel JL, Silva PJ, Rollyson MK, McIntush EW (2000). Mechanisms controlling the function and life span of the Corpus luteum. Physiol Rev.

[4] Tomac J, Cekinovć D, Arapović J (2011). Biology of the corpus luteum. Period Biol.

[5] Devoto L, Fuentes A, Kohen P, Cespedes P, Palomino A, Pommer R (2009). The human corpus luteum: life cycle and function in natural cycles. Reprod Endocrinol.

[6] Bergers G, Benjamin LE (2003). Angiogenesis: tumorigenesis and the angiogenic switch. Nat Rev Cancer.

[7] Julien-Flores V, Dorseuil O, Romero F, Letourner S, Saragoti R, Berger A (1995). Bridging Ral GTPase to rho pathways. J Biol Chem.

[8] Cantor SB, Urano T, Feig LA (1995). Identification and characterization of Ral-binding protein 1, a potential downstream target of Ral GTPases. Mol Cell Biol.

[9] Singhal SS, Yadav S, Drake K, Singhal J, Awasthi S (2008). Hsf-1 and POB1 induce drug sensitivity and apoptosis by inhibiting Ralbp1. J Biol Chem.

[10] Singhal SS, Wickramarachchi D, Yadav S, Singhal J, Leake K, Vatsyayan R (2011). Glutathione-conjugate transport by RLIP76 is required for Clathrin-dependent endocytosis and chemical carcinogenesis. Mol Cancer Ther.

[11] Wang W, Liu J, Qi J, Zhang J, Zhu Q, Qin C (2016). RLIP76 decreases apoptosis through Akt/mTOR signaling pathway in gastric cancer. Oncol Rep.

[12] Zhang Y, Song X, Gong W, Zhu Z, Liu X, Hou Q (2015). RLIP76 blockade by siRNA inhibits proliferation, enhances apoptosis, and suppresses invasion in HT29 Colon Cancer cells. Cell Biochem Biophys.

[13] Fraser HM, Wulff C (2001). Angiogenesis in the primate ovary. Reprod Fertil Dev.

[14] Gentry LR, Martin TD, Reiner DJ, Der CJ (2014). Ral small GTPase signaling and oncogenesis: more than just 15 minutes of fame. Biochim Biophys Acta Mol Cell Res.

[15] Awasthi YC, Sharma R, Yadav S, Dwivedi S, Sharma A, Awasthi S (2007). The non-ABC drug transporter RLIP76 (RALBP-1) plays a major role in the mechanisms of drug resistance. Current Drug Metab.

[16] Goldfinger LE, Lee S (2013). Emerging treatments in lung cancer - targeting the RLIP76 molecular transporter. Lung Cancer Targets Ther.

[17] Yamaguchi A, Urano T, Goi T, Feig LA (1997). An eps homology (EH) domain protein that binds to the Ral-GTPase target, RalBP1. J Biol Chem.

[18] Ikeda M, Ishida O, Hinoi T, Kishida S, Kikuchi A (1998). Identification and characterization of a novel protein interacting with Ral-binding protein 1, a putative effector protein of Ral. J Biol Chem.

[19] Koshiba S, Kigawa T, Iwahara J, Kikuchi A, Yokoyama S (1999). Solution structure of the Eps15 homology domain of a human POB1 (partner of RalBP1). FEBS Lett.

[20] Jullien-Flores V, Mahé Y, Mirey G, Leprince C, Meunier-Bisceuil B, Sorkin A (2000). RLIP76, an effector of the GTPase Ral, interacts with the AP2 complex: involvement of the Ral pathway in receptor endocytosis. J Cell Sci.

[21] Awasthi S, Cheng JZ, Singhal SS, Saini MK, Pandya U, Pikula S (2000). Novel function of human RLIP76: ATP-dependent transport of glutathione conjugates and doxorubicin. Biochemistry..

[22] Awasthi S, Cheng JZ, Singhal SS, Pandya U, Sharma R, Singh SV (2001). Functional reassembly of ATP-dependent xenobiotic transport by the N- and C-terminal domains of RLIP76 and identification of ATP binding sequences. Biochemistry..

[23] Vatsyayan R, Lelsani PCR, Awasthi S, Singhal SS (2010). RLIP76: a versatile transporter and an emerging target for cancer therapy. Biochem Pharmacol.

[24] Cheng JZ, Sharma R, Yang Y, Singhal SS, Sharma A, Saini MK (2001). Accelerated metabolism and exclusion of 4-Hydroxynonenal through induction of RLIP76 and hGST5.8 is an early adaptive response of cells to heat and oxidative stress. J Biol Chem.

[25] Hu Y, Mivechi NF (2003). HSF-1 interacts with Ral-binding protein 1 in a stress-responsive, multiprotein complex with HSP90 in vivo. J Biol Chem.

[26] Kashatus DF, Lim KH, Brady DC, Pershing NL, Cox AD, Counter CM (2011). RALA and RALBP1 regulate mitochondrial fission at mitosis. Nat Cell Biol.

[27] Lebreton S, Boissel L, Iouzalen N, Moreau J (2004). RLIP mediates downstream signalling from RalB to the actin cytoskeleton during Xenopus early development. Mechanisms Dev.

[28] Goldfinger LE, Ptak C, Jeffery ED, Shabanowitz J, Hunt DF, Ginsberg MH (2006). RLIP76 (RalBP1) is an R-Ras effector that mediates adhesion-dependent Rac activation and cell migration. J Cell Biol.

[29] Fenwick RB, Campbell LJ, Rajasekar K, Prasnnan S, Nietlispach D, Camonis J (2010). The RalB-RLIP76 complex reveals a novel mode of ral-effector interaction. Structure..

[30] Singhal SS, Awasthi YC, Awasthi S (2006). Regression of melanoma in a murine model by RLIP76 depletion. Cancer Res.

[31] Singhal SS, Singhal J, Yadav S, Sahu M, Awasthi YC, Awasthi S (2009). RLIP76: a target for kidney cancer therapy. Cancer Res.

[32] Singhal SS, Singhal J, Yadav S, Dwivedi S, Boor PJ, Awasthi YC (2007). Regression of lung and colon cancer xenografts by depleting or inhibiting RLIP76 (ral-binding protein 1). Cancer Res.

[33] Awasthi S, Singhal SS, Singhal J, Cheng JZ, Zimniak P, Awasthi YC (2003). Role of RLIP76 in lung cancer doxorubicin resistance: II. Doxorubicin transport in lung cancer by RLIP76. Int J Oncol.

[34] Stuckler D, Singhal J, Singhal SS, Yadav S, Awasthi YC, Awasthi S (2005). RLIP76 transports vinorelbine and mediates drug resistance in non-small cell lung cancer. Cancer Res.

[35] Singhal SS, Sehrawat A, Sahu M, Singhal P, Vatsyayan R, Lelsani PCR (2010). Rlip76 transports sunitinib and sorafenib and mediates drug resistance in kidney cancer. Int J Cancer.

[36] Sharma R, Singhal SS, Wickramarachchi D, Awasthi YC, Awasthi S (2004). RLIP76 (RALBP1)-mediated transport of leukotriene C4 (LTC4) in cancer cells: implications in drug resistance. Int J Cancer.

[37] Singhal SS, Yadav S, Singhal J, Drake K, Awasthi YC, Awasthi S (2005). The role of PKCα and RLIP76 in transport-mediated doxorubicin-resistance in lung cancer. FEBS Lett.

[38] Margutti P, Matarrese P, Conti F, Colasanti T, Delunardo F, Capozzi A (2008). Autoanitbodies to the C-terminal subunit of RLIP76 induce oxidative stress and endothelial call apoptosis in immune-mediated vascular diseases and atherosclerosis. Blood..

[39] Awasthi S, Singhal SS, Yadav S, Singhal J, Drake K, Nadkar A, Zajac E (2005). RLIP76 is a major determinant of radiation sensitivity. Cancer Res.

[40] Awasthi S, Singhal SS, Singhal J, Yang Y, Zimniak P, Awasthi YC (2003). Role of RLIP76 in lung cancer doxorubicin resistance: III. Anti-RLIP76 antibodies trigger apoptosis in lung cancer cells synergistically increase doxorubicin cytotoxicity. Int J Oncol.

[41] Singhal SS, Yadav S, Singhal J, Zajac E, Awasthi YC, Awasthi S (2005). Depletion of RLIP76 sensitizes lung cancer cells to doxorubicin. Biochem Pharmacol.

[42] Singhal SS, Yadav S, Singhal J, Awasthi YC, Awasthi S (2006). Mitogenic and drug-resistance mediating effects of PKCα require RLIP76. Biochem Biophys Res Commun.

[43] Leake K, Singhal J, Nagaprashantha LD, Awasthi S, Singhal SS (2012). RLIP76 regulates PI3K/Akt signaling and chemo-radiotherapy resistance in pancreatic cancer. PLoS One.

[44] Smith SK (2001). Regulation of angiogenesis in the endometrium. Trends Endocrinol Metab.

[45] Brown MR, Blanchette JO, Kohn EC (2000). Angiogenesis in ovarian cancer. Best Pract Res Clin Obstet Gynaecol.

[46] Fraser HM, Lunn SF (2000). Angiogenesis and its control in the female reproductive system. Br Med Bull.

[47] Plendl J (2000). Angiogenesis and vascular regression in the ovary. Anat Histol Embryol.

[48] Lamalice L, Boeuf FL, Huot J (2007). Endothelial cell migration during angiogenesis. Cir Res.

[49] Naumov GN, Folkman J, Straume O, Akslen LA (2008). Tumor-vascular interactions and tumor dormancy. Apmis..

[50] Lee S, Wurtzel JGT, Singhal SS, Awasthi S, Goldfinger LE (2012). RALBP1/RLIP76 depletion in mice suppresses tumor growth by inhibiting tumor neovascularization. Cancer Res.

[51] Singhal SS, Roth C, Leake K, Singhal J, Yadav S, Awasthi S (2009). Regression of prostate cancer xenografts by RLIP76 depletion. Biochem Pharmacol.

[52] Lee S, Goldfinger LE (2014). RLIP76 regulates HIF-1 activity, VEGF expression and secretion in tumor cells, and secretome transactivation of endothelial cells. FASEB J.

[53] Wu Z, Owens C, Chandra N, Popovic K, Conaway M, Theodorescu D (2010). RalBP1 is necessary for metastasis of human Cancer cell lines. Neoplasia..

[54] Lee S, Wurtzel JGT, Goldfinger LE (2014). The RLIP76 N-terminus binds ARNO to regulate PI 3-kinase, Arf6 and Rac signaling, cell spreading and migration. Biochem Biophys Res Commun.

[55] O’shea JD, Rodgers RJ, D’occhio MJ (1989). Cellular composition of the cyclic corpus luteum of the cow. J Reprod Fertil.

[56] Stocco C, Telleria C, Gibori G (2007). The molecular control of corpus luteum formation, function, and regression. Endocr Rev.

[57] Robinson RS, Woad KJ, Hammond AJ, Laird M, Hunter MG, Mann GE (2009). Angiogenesis and vascular function in the ovary. Reproduction..

[58] Fraser HM, Wulff C (2003). Angiogenesis in the corpus luteum. Reprod Biol Endocrinol.

[59] Zhang Z, Yin D, Wang Z (2011). Contribution of hypoxia-inducible factor-1α to transcriptional regulation of vascular endothelial growth factor in bovine developing luteal cells. Anim Sci J.

[60] Ferrara N, Chen H, Davis-Smith T, Gerber HP, Nguyen TN, Peers D (1998). Vascular endothelial growth factor is essential for corpus luteum angiogenesis. Nat Med.

[61] Al-zi’abi MO, Watson ED, Fraser HM (2003). Angiogenesis and vascular endothelial growth factor expression in the equine corpus luteum. Reproduction..

[62] Kaczmarek MM, Kowalczyk AE, Waclawik A, Schams D, Ziecik AJ (2007). Expression of vascular endothelial growth factor and its receptors in the porcine Corpus luteum during the estrous cycle and early pregnancy. Mol Reprod Dev.

[63] Redmer DA, Dai Y, Li J, Charnock-Jones DS, Smith SK, Reynolds LP (1996). Characterization and expression of vascular endothelial growth factor (VEGF) in the ovine corpus luteum. J Reprod Fertil.

[64] Berisha B, Schams D, Rodler D, Pfaffl MW (2016). Angiogenesis in the ovary - the Most important regulatory event for follicle and Corpus luteum development and function in cow - an overview. J Vet Med Anat Histol Embryol.

[65] Zimmermann RC, Hartman T, Bohlen P, Sauer MV, Kitajewski J (2001). Preovulatory treatment of mice with anti-VEGF receptor 2 antibody inhibits angiogenesis in corpora lutea. Microvasc Res.

[66] Sugino N, Kashida S, Takiguchi S, Karube A, Kato H (2000). Expression of vascular endothelial growth factor and its receptors in the human Corpus luteum during the menstrual cycle and in early pregnancy. J Clin Endocrinol Metab.

[67] Ferrara N (2002). VEGF and the quest for tumour angiogenesis factors. Nat Rev Cancer.

[68] Liu L, Ning X, Han S, Zhang H, Sun L, Shi Y (2008). Hypoxia induced HIF-1 accumulation and VEGF expression in gastric epithelial mucosa cells: involvement of ERK1/2 and PI3K/Akt. Mol Biol.

[69] Fukuda R, Hirota K, Fan F, Jung YD, Ellis LM, Semenza GL (2002). Insulin-like growth factor 1 induces hypoxia-inducible factor 1-mediated vascular endothelial growth factor expression, which is dependent on MAP kinase and phosphatidylinositol 3-kinase signaling in colon cancer cells. J Biol Chem.

[70] Zhang Z, Yu D, Yin D, Wang Z (2011). Activation of PI3K/mTOR signaling pathway contributes to induction of vascular endothelial growth factor by hCG in bovine developing luteal cells. Anim Reprod Sci.

